# Physiotherapy postgraduate studies in South Africa: Facilitators and barriers

**DOI:** 10.4102/sajp.v73i1.335

**Published:** 2017-02-15

**Authors:** Saul Cobbing, Stacy Maddocks, Simoene Govender, Shuaib Khan, Mpilonhle Mbhele, Kareena Naidoo, Summaya Tootla, Claire Weston

**Affiliations:** 1 Department of Physiotherapy, University of KwaZulu-Natal, South Africa

## Abstract

**Aim:**

To investigate the facilitators and barriers to attaining a postgraduate physiotherapy degree in South Africa.

**Methods:**

A quantitative, cross-sectional design using an internet-based survey was employed. The population of the study included all qualified physiotherapists who had completed community service and who were on the South African Society of Physiotherapy e-mailing list at the time of the study.

**Results:**

In all, 425 valid responses were received. The study participants were predominantly white women with a mean age of 36.9 and the majority were working in private practice. A total of 20.5% of respondents had completed a master’s or doctoral degree in physiotherapy, while a further 13% of respondents were registered for a postgraduate degree in physiotherapy at the time of the study. Study participants who had obtained a postgraduate degree identified the same main barriers (namely cost/lack of financial support, family commitments and lack of time) and the same main facilitators (namely gaining of expertise, fulfilment of a personal goal and improvement of patient care) as participants who had not obtained a postgraduate degree. Participants who had not obtained a postgraduate degree were significantly more likely (*p* < 0.05) to report concerns regarding their own ability and a lack of motivation as barriers to further study.

**Conclusion:**

South African physiotherapists with and without a postgraduate degree reported common facilitators and barriers to pursuing postgraduate studies. In order to ensure that a greater number and diversity of physiotherapists see postgraduate studies as a worthwhile career option, stakeholders in health and education in both the South African public and private sectors need to be engaged to limit the barriers to postgraduate study and seek novel methods of making postgraduate study a more attractive option from a personal development and career perspective.

## Introduction

For over two decades, calls for more research in physiotherapy have been made in order to assist in developing a distinct identity for the profession (Robertson 1995). The World Confederation for Physical Therapy recently formalised its definition of the profession, stating that physiotherapy ‘provides services to individuals and populations to develop, maintain and restore maximum movement and functional ability throughout the lifespan’. The broader description of the profession includes research as a key principle on which future practice should be built (WCPT 2014). Fifteen years ago, the eminent Australian professor of physiotherapy, Jack Crosbie, stated that while physiotherapists generally endorse the need for evidence-based practice, they are involved in relatively little research specific to the profession (Crosbie 2000). Research, including in the often ignored area of physiotherapy education, is crucial to guide practice in South Africa (Mothabeng 2006). While there is little published evidence on the involvement of South African physiotherapists in formal research activities, this state of affairs would appear to remain unchanged in this country. One way of encouraging clinicians to conduct research is for them to enrol in postgraduate physiotherapy programmes. These programmes, which include master’s and doctoral (or PhD) degrees, involve a significant research component and may also involve elements of course work (CHE 2009).

The South African government has, for a number of years, prioritised the attainment of postgraduate qualifications across all disciplines. The South African PhD Project, an initiative of the National Research Foundation, has planned a five-fold increase in the number of doctoral graduates by 2025, in an effort to upskill the number of South Africans who can contribute to the country’s economic growth (NRF 2015). In 2007, South Africa only produced 26 doctorates per million of the total population, compared to 52 per million in Brazil (a comparable economy), 201 per million in the United States of America and 569 per million in Portugal (ASSAF 2010). While attrition or drop-out rates from PhD studies may be as high as 40% in South Africa, Mouton (2007) argues that this is similar to international norms and states that the real problem is that there are too few doctoral students in the South African higher education system. This is caused by a number of factors including inexperienced and overburdened supervisors, insufficient research preparation and inadequate institutional and national financial support for postgraduate students. Many tertiary institutions in South Africa now state a minimum requirement of a master’s degree in order to be considered for a permanent lecturing position (UKZN 2008), while Kenya recently increased its minimum requirement for a lecturer position to a PhD qualification (University World News 2014). In a predominantly clinically based profession like physiotherapy, this provides an obstacle to heads of academic departments who may want to employ individuals with extensive clinical expertise, but who have not been awarded a master’s or doctoral degree. In addition to this, it is imperative in post-apartheid South Africa that physiotherapy departments should align themselves with policies of transformation (Ramklass 2009). This is crucial if the profession is to contribute to redressing the current reality in South Africa where knowledge production has predominantly been the domain of white men. The participation of black South African men and women in postgraduate studies is inadequate (Badat 2010).

Statistics on the growth rate of graduates doing postgraduate studies in South Africa indicate that health science master’s graduates have the lowest percent of annual growth rate of 5.2%. For doctoral studies, however, health science graduates achieve the second highest growth rate of 9.5% per annum, after the social sciences (CHE 2009). This appears to indicate that while relatively few health science graduates graduate from master’s programmes, those who do so are more likely to also complete their PhD. There is no specific evidence, however, on the take-up of postgraduate studies among physiotherapists in South Africa. International evidence in this regard is also limited. Sran and Murphy (2009) reported that 12% of a sample of 425 physiotherapists in British Columbia, Canada, had completed a postgraduate degree in physiotherapy, while a survey of 166 Australian physiotherapists revealed that 18.8% of respondents had completed postgraduate degrees (Grimmer-Somers, Lekkas & Nyland 2007). A benefit of more physiotherapists completing postgraduate studies is that individuals with a higher level of qualification are more likely to have a greater publication record (Frantz et al. 2010), thus adding more to the profession’s evidence base.

When considering the factors that may dissuade physiotherapists from pursuing postgraduate studies, it is instructive to explore the global evidence. Beeston, Rastall and Hoare (1998) suggested that physiotherapists choose not to pursue postgraduate studies because of the perception that these degrees are inherently academic and less relevant than the practical skills required to work in the clinical environment. This study, including 213 physiotherapists and managers in the United Kingdom, further revealed that physiotherapy managers did not regard master’s degrees as a valuable means of career advancement. Postgraduate studies are also seen as not being financially viable because of the fact that the undertaking of academic studies is often not supported with funding or study leave (Stathopoulos & Harrison 2003). Other barriers to pursuing postgraduate studies included inflexibility of available programmes (Gosling 1999), family commitments and geographical location (Sran & Murphy 2009). Glover, Bulley & Howden (2008) cite lack of time and personal motivation as well as minimal recognition in terms of increased pay or promotion as additional barriers. Similar factors related to time and recognition may explain the low publication rate by qualified physiotherapists, with only 6.9% of a sample of Kuwait-based physiotherapists reporting publications in academic journals (Hamzat & Amusat 2002). Murray and Newton (2008) propose that this low publication rate is, in part, because of the lack of training on academic writing in both undergraduate and postgraduate physiotherapy programmes.

The above information presents a paradox of sorts. The profession of physiotherapy seems to be committed to growing the evidence base underpinning the provision of clinical services. Postgraduate studies appear to be the most efficient means of achieving a growth in this profession-specific evidence. However, globally there is a reluctance by physiotherapists to pursue postgraduate studies, based on a number of reported barriers. This is mirrored by a relatively low publication output by qualified physiotherapists, which will in turn have a negative effect on the desired evidence base. What is not known is how many South African physiotherapists are currently pursuing postgraduate studies. The evidence discussed above emanates primarily from international studies, contrasting with the dearth of information related to the uptake of postgraduate studies by physiotherapists in South Africa. The aim of this study was to investigate the potential facilitators and barriers to their pursuit of postgraduate studies. It is hoped that the results of this study may be of benefit to policy makers in South African universities and health service providers, as well as to physiotherapists considering pursuing postgraduate studies.

## Methodology

### Design

A quantitative, cross-sectional design using an internet-based survey (see Appendix 1) was employed for this research.

### Study population and sample

The study population included all South African qualified physiotherapists who had completed community service as well as foreign-qualified physiotherapists residing in South Africa, all of whom were members of the South African Society of Physiotherapy (SASP) and on their e-mailing list at the time of the study. The study sample included all those who responded within the stipulated data collection period. Individuals who had not completed an undergraduate degree in physiotherapy or had not completed their community service year by the end of 2014 were excluded from this study.

### Data collection tool

A self-developed survey informed by the literature was utilised in this study. This survey (consisting of 17 questions) was formulated on Google forms including questions related to demographic information, qualification profile, attainment of a postgraduate degree, perceived barriers towards obtaining a postgraduate degree and facilitators towards pursuing a postgraduate degree. Permission to e-mail the study information and survey to the study population was granted by the chairperson of the SASP. The survey was sent out via e-mail by the SASP on behalf of the researchers to all qualified physiotherapists on the SASP e-mailing list. Comprehensive information related to the study was included in this e-mail including the motivation for the study and an outline of the study aims and objectives. A link to the survey was included in this e-mail and informed consent had to be accepted before continuing with the survey. Data were collected over a period of 10 weeks, and in the eighth week, a follow-up e-mail was sent out, reminding the study population to respond if they had not already done so.

In order to improve the content validity of the survey, a pilot survey was sent to 29 community service physiotherapists who had graduated from the University of KwaZulu-Natal in 2014. Thirteen responses were received and minor modifications and improvements were made to the survey.

### Data analysis

Information from the surveys was captured by Google forms and copied onto a Microsoft Excel spreadsheet where the data were summarised and incomplete responses were deleted. Data were analysed using SPSS version 22. Descriptive statistics (including mean, range and percentages) were calculated for demographic data. The number of responses (and percentages) for each of the barriers and facilitators to further study was calculated for two distinct groups – participants without a postgraduate degree and participants with a postgraduate degree. The Pearson Chi-Square test was employed to ascertain whether there was a significant association between the group (either with or without a postgraduate degree) and a specific response (facilitator or barrier) on the survey. The level of significance was set at *p* = 0.05.

### Ethical considerations

Ethical approval was granted by the University of KwaZulu-Natal’s School of Health Science’s Ethics Committee (Ref: SHSEC014/15).

## Results

### Sociodemographic and qualification profile

The survey was sent, via e-mail, to 4535 physiotherapists on the SASP e-mailing list. A total of 442 e-mail responses were received. This represents an overall response rate of 9.7%. Eighteen participants did not meet the study inclusion criteria, resulting in 425 responses being included for analysis. The mean age of participants was 36.9 years and 89% of participants were female. The majority (78.1%) of participants were working in private practice, while 9.4% worked for the South African Department of Health (DOH) and 9.3% worked in tertiary or primary education. A minority (20.5%) of participants had completed a postgraduate degree in physiotherapy. A majority (79.5%) of participants had only completed a bachelor’s degree in physiotherapy, with 13% of these participants registered for postgraduate degree at the time of the study. Almost half of the participants expressed interest in doing a postgraduate degree in physiotherapy, while 26% were disinterested. Table 1 displays the full demographic and qualification profile of all 425 study participants.

**TABLE 1:** Sociodemographic data of study participants (*n* = 425).

Characteristic	Value
Age (mean years)	36.9 (range, 22–76)
Gender	89% female, 11% male
Race (%)	
Black	7.4
Coloured	6.1
Indian	7.5
White	76.6
Other	2.4
Employment sector (%)	
Private (full-time or locum)	78.1
Public (Department of Health)	9.4
Education (tertiary)	7.2
Unemployed or other	3.3
Education (school)	1.6
Full-time student	0.4
University of undergraduate degree (%)	
University of Witwatersrand	19.1
University of Pretoria	18.2
University of the Free state	15.3
University of Cape Town	15.1
Stellenbosch University	13.4
University of KwaZulu-Natal	6.6
University of the Western Cape	4.7
University of Limpopo	2.4
Other	5.2
Predominant area of practice (%)	
Orthopaedics	28.2
Sports	16.1
Neurology	12.3
Cardiorespiratory	9.3
Paediatrics	8.8
General medicine	8.6
Community health	3.3
Women’s health	2.6
Education	2.6
Other	8.2
Highest level of qualification (%)	
Bachelor’s degree	79.5
Master’s: course work	10.2
Master’s: research	6.8
Doctorate: research	3.3
Doctorate: coursework	0.2
Registered for postgraduate physiotherapy degree (%)	13.2
Interested in doing postgraduate physiotherapy degree (%)	
Yes	45.4
No	25.9
Maybe	28.7

*Source*: Authors’ own work

### Facilitators and barriers

The results of this study have been divided into two categories; namely physiotherapists without a postgraduate degree and physiotherapists with a postgraduate degree. Table 2 illustrates the barriers to postgraduate study reported by both physiotherapists without a postgraduate degree and those who had attained a postgraduate degree in physiotherapy. Table 3 illustrates the facilitators reported by these two groups. Both these tables display the number (*n*) of responses for each barrier or facilitator listed in the study survey, as well as this number represented as a percentage of the total number of respondents from each group. Tables 2 and 3 also show the association between the two groups and each response, with the respective *p*-values calculated using the Pearson Chi-Square test. The results show that participants without a postgraduate degree indicated the same three main barriers as those highlighted by the participants with postgraduate qualifications, namely cost/lack of financial support, lack of time and family commitments (in that order in both groups). Similarly, both groups indicated the same three most prevalent facilitating factors, namely development of expertise, fulfilment of a personal goal and improvement of patient care (with the development of expertise first in the non-postgraduate group and the fulfilment of a personal goal first in the postgraduate group). The only significant difference between the two groups was related to two of the proposed barriers; with participants with a degree significantly less likely to name concerns regarding their ability and a lack of motivation as a barrier to further study. There were no significant differences between the two groups related to potential facilitators to further study.

**TABLE 2:** Barriers to postgraduate study.

Variables	Without a PG degree (*n* = 338)		With a PG degree (*n* = 87)		Association (*p*)
	*n*	%	*n*	%	
Age	29	8.6	4	4.6	0.216
Concerns regarding ability	24	7.1	1	1.1	0.035*
Cost	58	17.2	10	11.5	0.199
Family commitments	59	17.5	12	13.0	0.414
Geographic location	26	7.7	2	2.3	0.071
Lack of supervision	21	6.2	6	6.9	0.816
Lack of employer support	31	9.2	6	6.9	0.502
Lack of motivation	18	5.3	0	0	0.028*
Lack of time	59	17.5	13	14.9	0.577
Negative past experiences	19	5.6	2	2.3	0.202
Prefer other studies	36	10.7	5	5.7	0.167
Other	37	10.9	6	6.9	0.264

*Source*: Authors’ own work

* *p* < 0.05.

**TABLE 3:** Facilitators to postgraduate study.

Variables	Without a PG degree (*n* = 338)		With a PG degree (*n* = 87)		Association ( *p*)
	*n*	%	*n*	%	
Compulsory requirement	15	4.4	2	2.3	0.364
Development of expertise	50	14.8	11	12.6	0.610
Financial incentive	27	8.0	2	2.3	0.101
Personal goal	42	12.4	12	13.8	0.733
Improve patient care	50	14.8	11	12.6	0.252
Improved job opportunities	29	8.6	6	6.9	0.611
Increased income	38	11.2	3	3.4	0.008
Increased job satisfaction	26	7.7	8	9.2	0.645
Move to a different field	15	4.4	3	3.4	0.683
Prestige attached	24	7.1	4	4.6	0.401
Other	11	3.3	5	5.7	0.276

*Source*: Authors’ own work

## Discussion

According to Robertson (1995), research is expected to provide the unique knowledge base of any discipline, which is required for the performance of professional roles. The physiotherapy knowledge base, both in South Africa and globally, can be grown by the uptake of postgraduate qualifications by physiotherapists in this country. This study has reported on the qualification profile of registered physiotherapists in South Africa and has further investigated the potential facilitators and barriers towards physiotherapy postgraduate studies.

The response rate of 9.7% appears to be poor in comparison to a systematic review of 490 studies utilising surveys, conducted by Baruch and Holtom (2008), which showed an average 52.7% response rate for all types of surveys and a slightly higher response rate (55.5%) for Internet-mailed surveys. However, studies surveying physiotherapists have exhibited far lower response rates than this average. An Australian survey (McKiernan, Chiarelli & Warren-Forward 2011) which received 99 responses from qualified physiotherapists had a 20.5% response rate, while two Canadian surveys (Francis et al. 2012; Sran & Murphy 2009) reported response rates of 17% (425 respondents) and 27% (114 respondents), respectively. A possible reason for the low response rates among physiotherapists may be that physiotherapy is a clinically based profession and physiotherapists spend minimal time during the work day at their desk or behind a computer. That being said, this study yielded a markedly lower response rate than the three physiotherapy surveys described above. This lower response rate may be explained by the fact that, for confidentiality reasons, the survey was administered by a third party (the SASP) and the researchers were unable to see which e-mails had not been delivered. Furthermore, sending one e-mail to such a large number of individual addresses (4535) would have resulted in a significant number of potential respondents not actually reading the mail due to it going straight into their spam or bulk mail folder. The expanded use of spam or bulk mail folders (for e-mails sent to large numbers of recipients) may explain the gradual downward decline in overall response rates to e-mail surveys from above 60% in 1986 (when e-mail surveys were first used) to below 24% post-2000, as reported in a systematic review of e-mail surveys by Sheehan (2001).

The majority (89%) of participants in this study were women, which is similar to another South African study surveying Gauteng-based SASP members, which had 92% female respondents (Mostert-Wentzel et al. 2012). Two international studies surveying qualified physiotherapists also showed a significant majority of female respondents of 79% (McKiernan et al. 2011) and 99% (Francis et al. 2012), although it should be pointed out that this latter study surveyed physiotherapists with a specific interest in women’s health. This high female response rate across physiotherapy-focused studies is likely because of the overall profession being a female-dominant discipline in most countries (Higgs & Elizabeth Ellis 2001), although there is evidence that in recent years more men are entering the profession (Schofield & Fletcher 2007).

The results of this study showed that 17.0% of participants had completed a master’s degree in physiotherapy with a further 3.5% of participants stating that their highest level of qualification was a doctoral degree (all but one participant having completed research doctorates). The number of participants with a coursework master’s was double that of those with a master’s in research. According to Jull and O’Sullivan (2006), a coursework master’s offers benefits including acquiring advanced diagnostic and management skills and improved patient outcomes, presenting a possible reason as to why coursework is preferred over research. Clinical master’s qualifications can also assist in improving physiotherapists’ patient-centred practice as well as increase their capability to learn in, and from, practice (Petty, Scholes & Ellis 2010). Unfortunately, in South Africa, Swanepoel (2010) highlights the huge discrepancy between the increased demand for coursework master’s programmes and the lower level of importance and funding attached to these options by many tertiary institutions.

The total of 20.5% of participants in this study with a postgraduate degree in physiotherapy is higher than that found in two international studies, with Sran and Murphy (2009) reporting a 12% postgraduate rate among Canadian physiotherapists and Grimmer-Somers et al. (2007) reporting an 18.8% rate among Australian physiotherapists. Both these studies used survey protocols. The only previous survey conducted related to postgraduate physiotherapy studies in South Africa was conducted 25 years ago (Irwin-Carruthers 1991). This study found that 6% (25 out of 399) of South African physiotherapists responding to this survey had a postgraduate degree. The significant increase (of over 14%) in the proportion of South African physiotherapists holding a postgraduate qualification is certainly a positive development; however, it cannot be concluded that this ratio is as high as 20.5% across the country. Possible reasons for this relatively high number of participants having postgraduate degrees could be because of participants being recruited via the SASP e-mailing list, which may not be representative of the entire population of South African physiotherapists, as well as the fact that physiotherapists with postgraduate degrees (because of their increased interest in a study of this nature) may have been more likely to respond to this survey. However, it should be noted that a master’s-level study conducted with Gauteng-based public sector physiotherapists revealed that 26.3% (20 out of 76) respondents had a postgraduate qualification in physiotherapy (Rakgokong 2008).

An interesting finding in this study was that participants with or without a postgraduate degree identified the same three most common facilitators and barriers to pursuing postgraduate studies in physiotherapy. The common facilitating factors identified by both these groups were development of expertise, fulfilment of personal goals and improved patient care. These results were similar to a study done in the United Kingdom, exploring factors influencing physiotherapist decisions to study at master’s level, where the consistent facilitating factor for postgraduate studies among physiotherapists was being able to develop professionally or personally (Glover et al. 2008). It was encouraging to see that 13.2% of the study participants are currently registered for a postgraduate degree in physiotherapy. This shows that the facilitating factors are seen to outweigh the barriers for these individuals. According to Stathopoulos and Harrison (2003), physiotherapists see a relevance between academic master’s education and clinical practise as these programmes develop expertise and result in graduates being better equipped and more skilled clinically. Similarly, Beeston et al. (1998) reported that physiotherapists were more likely to pursue academic qualifications that placed a greater emphasis on the acquisition of clinical skills.

The three most common barriers reported by participants with or without a postgraduate degree were cost/lack of financial support, lack of time and family commitments. Interestingly, time, family commitments and lack of financial support (in that order) were also stated as the three major barriers to further study by clinicians looking to participate in a clinical master’s programme in Canada (Sran & Murphy 2009). The barrier of family commitment in this study may be because the majority of the participants were women, thus possibly having the added responsibility of caring for young children and elderly parents. Time was also stated as the most common factor militating against research participation in a survey of 87 Kuwait-based hospital physiotherapists (Hamzat & Amusat 2002), highlighting the universal problem of pursuing work and research goals simultaneously. In a study of senior physiotherapists in the United Kingdom, it was revealed that the majority of the respondents would have been more likely to consider studying for a higher degree in physiotherapy, if it were recognised in terms of better pay or promotion (Beeston et al. 1998). This further highlights the need for incentives to be implemented in order to encourage the pursuit of a postgraduate degree in physiotherapy.

There were only two significant differences between the two groups; both related to barriers to further study. A concern about one’s own ability was chosen as a barrier by 24 of the participants without a postgraduate qualification in this study, as compared to only one of the participants who had been awarded a postgraduate degree, representing a significant difference (*p =* 0.035) between the groups. In a qualitative study, conducted in Australia (Glover et al. 2008), physiotherapists reported additional barriers when deciding to study at master’s level, including a fear of failure and a lack of belief in their own academic ability. This finding is supported by the results in this study, suggesting that the participants with postgraduate degrees had grown in confidence on the successful completion of these qualifications. The other significant difference between the groups was related to lack of motivation, with 18 of the participants without a degree reporting this as a barrier, compared to none of the participants with a degree (*p* = 0.028). This is an interesting finding, suggesting that the participants with a postgraduate degree remain motivated to study further.

## Limitations

Caution should be taken when generalising the results of this study to the entire population of South African physiotherapists, because of the fact that only individuals on the SASP e-mailing list were invited to participate in this study. The survey method was employed for practicality and cost reasons, but this in itself may have resulted in the low response rate, because of members not having access to their mail during the work day or not having the time to respond via this medium. Furthermore, e-mails were mediated via SASP for confidentiality reasons. This may have resulted in a possible lack of accuracy because of the researchers being unaware of the rate of delivery error as well as being unable to regularly send follow-up e-mails to respondents. The survey method in itself is a limitation, as it does not allow for a more nuanced interrogation of participants’ responses.

## Recommendations

Further research involving all physiotherapists in South Africa needs to be pursued in order to obtain results that can be made more generalised to the entire country’s physiotherapy population. Interdisciplinary research with other health science professionals should be conducted to investigate if common barriers and facilitators are seen in other disciplines. Indeed, Louw et al. (2007) believes that the interest in research among South African physiotherapists is increasing for both academics and clinicians, with a clear interest from physiotherapists in conducting multidisciplinary collaborative research. Qualitative research methods would also add more depth and value to the information obtained from quantitative methods such as surveys. To address the under-representation of black students in physiotherapy programmes at many South African universities (Mbambo 2004), every effort should be made to improve the access and appeal of postgraduate physiotherapy programmes to this population group. These changes need to be instituted at the undergraduate level and sustained and enhanced to ensure that black students are encouraged to continue with postgraduate studies in physiotherapy. In order to address the barriers faced by physiotherapists considering the pursuit of postgraduate qualifications, incentives such as salary increases or promotions, fee reduction, or study leave need to be put in place by academic institutions and the DOH to facilitate the pursuit of postgraduate degrees. Jull (2006) argues that physiotherapists with postgraduate qualifications must be remunerated appropriately, an eventuality that can be achieved by investment from private and public stakeholders who benefit directly from the advanced skills of these graduates. Finally, physiotherapy departments in South African universities should offer more coursework master’s options to reflect the increased demand by professionals, while the universities themselves should ensure that greater funding and academic status are attached to these programmes.

## Conclusion

The majority of this study’s participants only held a bachelors degree in physiotherapy, with approximately one fifth of respondents having completed master’s and doctorate degrees. South African physiotherapists with or without a postgraduate degree reported common facilitators and barriers to pursuing postgraduate studies. Common facilitators were reported to be development of expertise, fulfilment of a personal goal and improvement of patient care, while common barriers included **cost**/lack of financial support, family commitments and lack of time. A lack of financial incentives towards doing a postgraduate degree in terms of salary, promotion or medical aids were also identified, and these incentives, as well as other novel programme choices, need to be implemented to encourage the pursuit of postgraduate studies by South African physiotherapists. These barriers need to be addressed by academic institutions, in collaboration with prospective students and other stakeholders such as the DOH, to ensure that more physiotherapists see postgraduate studies as a viable and attractive career option.
